# Supplementary material for: Crown ether decorated silicon photonics for safeguarding against lead poisoning
Source: Nat Commun. 2024 May 14;15:3820. doi: 10.1038/s41467-024-47938-6 (PMC11094186; doi:10.1038/s41467-024-47938-6)
Supplement: Supplementary file 1 — Supplementary Information [file 41467_2024_47938_MOESM1_ESM.pdf]

Supporting Information

Crown ether decorated silicon photonics for safeguarding against lead poisoning

Luigi Ranno[1,†], Yong Zen Tan[2,†], Chi Siang Ong[2], Xin Guo[3], Khong Nee Koo[4], Xiang Li[3], Wanjun Wang[3], Samuel Serna[1], Chongyang Liu[5], Rusli[3], Callum G. Littlejohns[6], Graham T. Reed[6], Juejun Hu[1], Hong Wang[3] and Jia Xu Brian Sia[1,3,*]

[1]*Department of Materials Science & Engineering, Massachusetts Institute of Technology, Cambridge, M.A., USA*

[2]*Fingate Technologies Pte Ltd, 8 Cleantech Loop, #06-65, 637145, Singapore*

[3]*School of Electrical and Electronic Engineering, Nanyang Technological University, 50 Nanyang Avenue, 639798, Singapore*

[4]*Vulcan Photonics SDN. BHD. D-11-08, Menara Suezcap, 1 KL Gateway, No. 2, Jalan Kerinchi, Kampung Kerinchi, 59200, Kuala Lumpur, Malaysia*

[5]*Temasek Laboratories, Nanyang Technological University, 50 Nanyang Avenue, 637553, Singapore*

[6]*Optoelectronics Research Centre, University of Southampton, Southampton SO17 1BJ, UK*

[†]*These authors contributed equally*

**Corresponding author: jiaxubrian.sia@ntu.edu.sg*

Table of Contents

Supplementary Note 1: Perspectives on Emerging Pb^{2+} Detection Technologies for Widespread Implementation 3

Supplementary Note 2: Sensor Temporal Dependence analyzed through XPS 8

Supplementary Note 3: Exploded-view schematic representation of the Pb^{2+} ion sensor assembly 9

Supplementary Note 4: Definition of FoM for the Optimization of Surface Sensitivity 10

Supplementary Note 5: Derivation of Equation 3 in the main text, relating designed loss (water absorption) with optimal splitter power ratios 13

Supplementary Note 6: EDX analysis prior and after functionalization, and after Pb^{2+} exposure and analyte flush and dry (N_2) 16

Supplementary Note 7: Detection of Sn^{2+} through XPS analysis after functionalization 17

Supplementary Note 8: Details on chip fabrication 19

Supplementary Note 9: Measured optical spectrum of the photonic sensor when the sensing region is exposed to air 21

Supplementary Note 10: Mathematical relationship between sensor resonant shift and exposed Pb^{2+} concentrations 22

Supplementary Note 11: Definition of crown ether decorated photonic sensor lower and upper detection limit 23

Supplementary Note 12: Sensor Reusability 24

Supplementary Note 13: Pb^{2+} ion photonic sensor selectivity test against Na^{+}, Mg^{+}, Cu^{+}, Sn^{+2}, Ca^{+2}, Fe^{2+} 28

Supplementary Note 14: ICP-MS elemental analysis of environmentally related scenarios; tap lake and sea water 29

Supplementary Note 15: Pb^{2+} photonic sensor performance in tap, lake and sea water, where the Pb^{2+} concentrations are synthetically increased by 15 ppb verified by ICP-MS 34

Supplementary Note 1: Perspectives on Emerging Pb^{2+} Detection Technologies for Widespread Implementation

This section aims to indicate a perspective of this work and alternative technologies (fluorescence[1–4], SWASV electrochemical[5–8], colorimetric (quantitative)[9,10], fiber-based[11], ICP-MS[12]/OES[13]), targeting the pervasive adoption of Pb^{2+} environmental sensing. Supplementary Table 1 seeks to provide an overview of the crown ether decorated silicon photonic Pb^{2+} sensing platform with well-established technologies. Notably, there have been remarkable developments in SWASV electrochemical [5–8], and fluorescence-based [1–4] Pb^{2+} sensors, where highly-selective, ppb-scale detection in environmentally-related samples has been demonstrated. The availability of compact analytical instruments can facilitate the mobile implementation of these sensors through discrete systems. For instance, handheld potentiometers[14] for SWASV electrochemical sensors, and micro-photospectrometer[15] for fluorescence sensors. It bears mentioning, however, that the significant costs of these analytical instruments will limit essential widespread sensor adoption, specifically in developing nations, where the impact of Pb^{2+} poisoning is the most significant[16,17]. The application of optics in Pb^{2+} detection represents an emerging technology, where the demonstration of the fiber-based Pb^{2+} sensor have indicated the possibility of cost reductions in portable detection via the application of the fiber interrogator as the analytical instrument[11]. However, all of the abovementioned detection schemes represents discrete applied systems, and as such might not possess the scalability, costs and the scale of implementation that an integrated silicon system can offer[18–20]. A key example that indicates the advantages of such integrated systems can be observed from the miniaturization of transistors, leading to the ubiquitous availability of personal computing. Furthermore, most of the Pb^{2+} sensing technologies have indicated a fixed or limited pH range, which imply requirements in sample processing (Supplementary Table 1). Through the first development and demonstration of the crown ether decorated SiP platform,

this work addresses the realization of Pb^{2+} sensors within an integrated SiP platform, demonstrating highly-selective ppb-scale detection with a large detection dynamic range (1 - 62000 ppb), pH resilience (6 - 8), and indicating the capacity for deployment in environmentally related scenarios (tap water, lake water, sea water). The sensor pH operating range is commensurate with that of typical environmental conditions[21], and its detection dynamic range shows its capacity to be applied in a multitude of applications: from the monitoring of drinking water[22] to heavy industry (i.e., mining[23], smelting[24], battery manufacturing[24], effluent monitoring[24]).The demonstrated Pb^{2+} photonic sensing platform is compatible with waveguide-based analytical components (i.e., spectrometer[25]), already demonstrated within the SiP ecosystem. This leads to the potential full integration of Pb^{2+} detection, and subsequent analysis, all at the chip-scale. Furthermore, by leveraging on the economies of scale pertaining to silicon manufacturing, these sensor systems can be manufactured at low-costs, without compromises in performance[26–29]. These factors underscores the much-needed proliferation of low-cost, high performance Pb^{2+} chip-scale sensors to safeguard against widespread lead toxification in society.

Technology	Demonstrated Detection Range/LoD	Demonstrated Detection Accuracy	Demonstrated Ion Selectivity Against	Preprocessing Requirement	Environmentally Relevant Water Sources	In situ, real-time measurement	pH operating range	System integration	Scalability*	Reference
Crown-ether decorated silicon photonics platform	1 - 62000 ppb/ 0.882 ppb	RSD of 4.65 % at the 15 ppb (EPA limit[22])	Na^+, K^+, Mg^{2+}, Li^+, Zn^{2+}, Ca^{2+}, Fe^{3+}, Cu^{2+}, Al^{3+}, Sn^{2+}, Cd^{2+}, Pb^{4+}	No	Tap water, lake water, sea water	Real time (2 minutes)	6 - 8	Fully-integrated system	Leverages on highly-scalable silicon manufacturing and wafer-scale functionalization	This Work
Fluorescence sensor (G-quadruplex formation)	3.79 - 100 ppb/ 3.79 ppb	RSD of 5.81 % at reference Pb^{2+} concentration of 15 ppb	K^+, Na^+, Ag^+, Mg^{2+}, Ca^{2+}, Zn^{2+}, Mn^{2+}, Cu^{2+}, Ni^{2+}, Fe^{2+}, Hg^{2+}, Cd^{2+}, Fe^{3+}	Yes	No	25 minutes	8	Discrete systems	Limited scalability; costly sensor instrumentation	ref[1]
Fluorescence sensor (liquid crystals and aggregation induced emission luminogens)	4.144 - 20720 ppb/ 0.135 ppb	N.R.	Cu^{2+}, Zn^{2+}, Ag^+, Cd^{2+}, Mg^{2+}, Mn^{2+}, K^+	No	No	Real time	N.R.	Discrete systems	Limited scalability; costly sensor instrumentation	ref[2]
Florescence sensor (leadglow)	10 - 50 ppb/ 10 ppb	N.R.	Li^+, Na^+, K^+, Ca^{2+}, Mg^{2+}, Fe^{2+}, Co^{2+}, Ni^{2+}, Cu^{2+}, Zn^{2+}, Cd^{2+}, Mn^{2+}, Hg^{2+}, As^{2+}, Sn^{2+}	No	N.R.	Real time	4 - 10	Discrete systems	Limited scalability; costly sensor instrumentation	ref[3]
Fluorescence sensor (graphene quantum dots and nanoparticles)	10.36 - 828.8 ppb/ 3.46 ppb	N.A.	Mn^{2+}, Fe^{3+}, K^+, Hg^{2+}, Cu^{2+}, Mg^{2+}, Ca^{2+}, Zn^{2+}, Cd^{2+}, Ag^+	No	No	> 60 minutes	7.4	Discrete systems	Limited scalability; costly sensor instrumentation	ref[4]
Electrochemical SWASV sensors	2.28 - 1036 ppb/ 2.28 ppb	RSD of 4.16 - 4.55 % at reference Pb^{2+} concentration of Pb^{2+} 2900 ppb	Ni^{2+}, Cs^{2+}, Cr^{3+}, Co^{3+}, $SO4^{2-}$	Yes	Drinking water, tap water, leucine, typtophan, 3-bromo benzaldehyde, 3-	No, i.e., sample preprocessing required	4	Discrete systems	Limited scalability; costly sensor instrumentation	ref[5]

					hydroxy benzaldehyde					
Electrochemical SWASV sensors	5.18 - 103.6 ppb/ 1.368 ppb	RSD of 1.21 % at reference Pb^{2+} concentration of 100 ppb	Cd^{2+}, Cu^{2+}	Yes	Environmental water	No, i.e., sample preprocessing required	4.8	Discrete systems	Limited scalability; costly sensor instrumentation	ref[6]
Electrochemical SWASV sensors	1 - 20 ppb/ 0.3 ppb	RSD of 5.9 % at reference Pb^{2+} concentration of 10 ppb	Zn^{2+}, Cd^{2+}, Cu^{2+}	Yes	Tap water	No, i.e., sample preprocessing required	4.5	Discrete systems	Limited scalability; costly sensor instrumentation	ref[7]
Electrochemical SWASV sensors	20.72 - 435.1 ppb/ 0.808 ppb	RSD of 1.23 - 1.76 % at reference Pb^{2+} concentration of 100 ppb	Zn^{2+}, Fe^{3+}, Cd^{2+}, Ni^{2+}, Cu^{2+}, Na^{+}	Yes	Tap water, lake water, river water	No, i.e., sample preprocessing required	5	Discrete systems	Limited scalability; costly sensor instrumentation	ref[8]
Colorimetric sensors by Au Nanoparticles Assembled by DNAzymes	82.88 - 414.4 ppb/ N.R.	N.R.	Co^{2+}, Zn^{2+}, Cd^{2+}, Mn^{2+}, Ni^{2+}, Ca^{2+}, Mg^{2+}	N.R.	N.R.	Real time	N.R.	Discrete systems	Limited scalability; costly sensor instrumentation	ref[9]
Colorimetric sensors by Pb^{2+} responsive borate glass powder	70 - 2500 ppb/ 70 ppm with spectrometer, 400 ppm visually	N.A.	Ca^{2+}, Ba^{2+}, Mg^{2+}, Hg^{2+}, Cd^{2+}, Cu^{2+}, Co^{2+}, Cr^{3+}, Al^{3+}, Fe^{3+}	No	N.R.	Real time	N.R.	Discrete systems	Limited scalability; costly sensor instrumentation	ref[10]
Fiber optic sensor (L-glutathione-modified)	5 - 50 ppb/ 5 ppb	N.R.	Ca^{2+}, Cs^{2+}, Cu^{2+}, Fe^{2+}, Mg^{2+}, Mn^{2+}, Ni^{2+}, Zn^{2+}, Cd^{2+}	Might be needed	Tap water	Real time	6 - 7	Discrete systems	Limited scalability; costly	ref[11]
ICP-MS	< 1 ppt - 1000 ppm	RSD of 0.9 % at 19.63 ppb	All metal ions	Yes	Any sample is possible as long as it can be acid digested and diluted into aqueous phase	No, i.e., sample preprocessing required and need to be sent to a laboratory	< 7	Discrete systems	Limited scalability; costly	ref[30]
ICP-OES	10 ppb - 10000 ppm	RSD of 2.1 % at 50 ppb	All metal ions	Yes	Any sample is possible as long as it can be acid digested and	No, i.e., sample preprocessing required and	< 7	Discrete systems	Limited scalability; costly	ref[31]

					diluted into aqueous phase	need to be sent to a laboratory				

*Scalability refers to the compatibility of the technologies with large-scale manufacturing technologies (i.e., CMOS process), in addition to viability for full sensor system chip-scale integration (sensor and analytics[25]).

Supplementary Table 1 Comparison of the crown ether decorated silicon photonic Pb^{2+} sensing platform with well-established technologies.

Supplementary Note 2: Sensor Temporal Dependence analyzed through XPS

The temporal dependence of the crown ether decorated silicon photonic Pb^{2+} sensor is analyzed by the time taken for the Pb^{2+} ions to bind to the crown ether (DBTDA) functional layer. The extent of binding was assessed using XPS. Four sensor chips are separately exposed to Pb^{2+} in DI water (pH = 6.8), with reference concentrations of 100 ppb, where the exposure times are varied between 10, 60, 120, and 180 s. The Pb $4f_{5/2}$ and Pb $4f_{7/2}$ binding energies with regards to the respective samples are shown in Supplementary Fig. 1.It can be seen that the XPS signal strength increases from 10 s to 120 s, and remains stable after, as indicated by the sample where the exposure times is 180 s. Consequently, it can be concluded that an equilibrium between Pb^{2+} ions and the crown ether functional layer is reached at 120 s. To that effect, a analyte exposure time of 120 s is maintained throughout the manuscript.

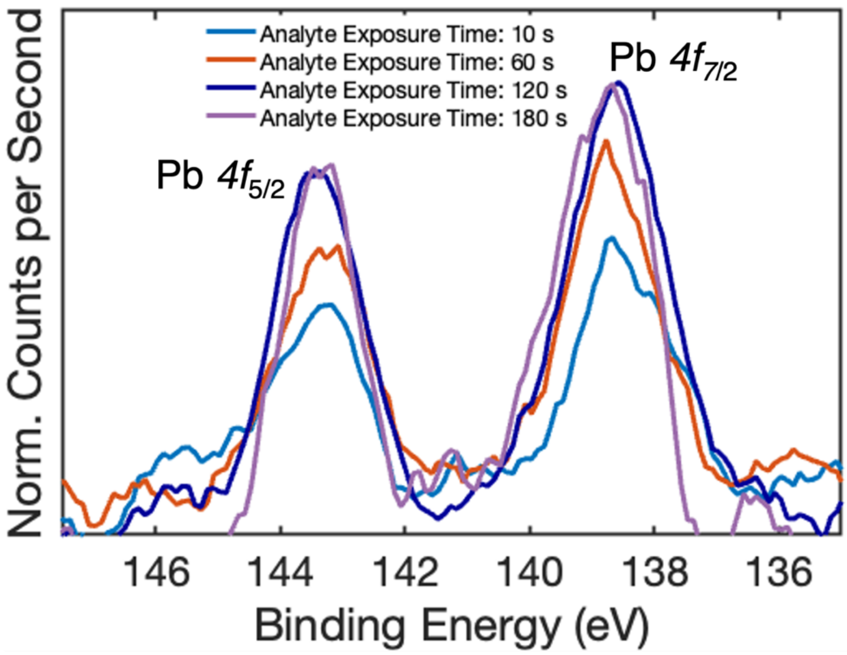

Supplementary Fig. 1 Sensor temporal dependence. Corresponding XPS spectrum measured from the photonic chip where the analyte comprises of 100 ppb of Pb^{2+} reference concentration in DI water. The exposure times are varied from 10, 60, 120, and 180 s. The pH of the analyte is maintained at 6.8 (see Methods).

Supplementary Note 3: Exploded-view schematic representation of the Pb^{2+} ion sensor assembly

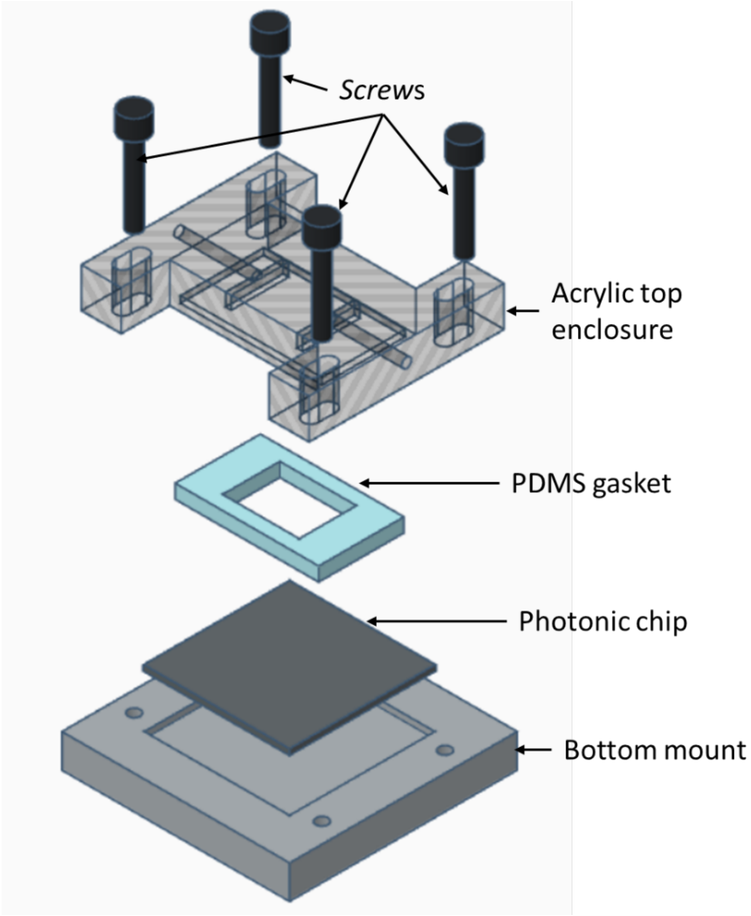

Supplementary Fig. 2. Exploded view representation of the Pb^{2+} ion sensor assembly, consisting of the bottom mount, photonic chip, PDMS gasket, acrylic top enclosure, and the screws, which are used to secure the assembly, and prevent microfluidic chamber leakage. The assembly is mounted on top of a TEC controller for thermal stabilization.

Supplementary Note 4: Definition of FoM for the Optimization of Surface Sensitivity

The cross section of a slot waveguide is illustrated in Supplementary Fig. 3a, with strip and slot width indicated; 20 nm of SiO_2 is deposited on the strip via ALD prior functionalization (Fig. 3a of the main text). For instance, the electric field distribution of the implemented slot waveguide, with strip and slot width of 240 nm is shown in Supplementary Fig. 3b. Higher surface sensitivity implies that power of the optical mode about the surface of the strip width will be higher. To that effect, the FoM, which considers the confinement factor, 10 nm from the waveguide surface with 20 nm of SiO_2 cladding, is derived from the following:

$$\mathrm{FoM} = \frac{\partial \mathrm{n}_{\mathrm{eff.}}}{\partial \mathrm{n}_{\mathrm{confine}}} = \frac{n_{\mathrm{g}}}{n_{\mathrm{confine}}} \frac{\int_{\mathrm{confine}} \varepsilon |\vec{\mathbf{E}}|^2 \mathrm{dA}}{\int \varepsilon |\vec{\mathbf{E}}|^2 \mathrm{dA}} \quad \text{(S4.1)}$$

$n_{eff.}$ and n_g are the effective index and group index of the optical mode respectively, $\vec{\mathbf{E}}$ is the electric field of the waveguide mode, $n_{confine}$ and $\boldsymbol{\varepsilon}$ are the material index and the dieletric permittivity of the medium. In the computation, the medium surrounding the waveguide is taken to be water (aqueous phase). The 2D integral in the numerator is to be taken about the 10 nm region which surrounds the waveguide and its cladding, while the integral at the denominator is considered about the entire space. The computed FoM of the slot waveguide is indicated in Fig. 2b of the main text. It is known that when the slot width is comparable to the exponential decay length of the fundamental eigenmode, optical power perpendicular to the high-index contrast interfaces is amplified [32–37]. This increases the surface sensitivity of the waveguide. As a comparison, the confinement factor (FoM) 10 nm about the surface of strip TE and TM waveguides with 20 nm of SiO_2 cladding was also computed. The cross-sectional illustration of the strip waveguide is illustrated in Supplementary Fig. 3c. The FoM of the strip waveguides at the fundamental TE and TM polarization are computed as a function of strip widths in Supplementary Fig. 3d. On the 220 nm SOI platform, at $\lambda = 1.55$ μm, the optimal waveguide width for surface sensing at the TE polarization is found to be 270 nm; the electric

field distribution of the waveguide mode is shown in Supplementary Fig. 3e. The FoM of TM strip waveguides is found to be lower than that of TE. This is because for TM polarization, the optical field is intensified at the top and bottom interfaces of the waveguide (Supplementary Fig. 3f)[38], unlike TE (Supplementary Fig. 3e), which is lateral [38]. The field at the bottom interface of the TM waveguide does not contribute to surface sensitivity. Furthermore, as the BOX has higher material refractive index than DI water, the field intensity at the bottom interface of the TM strip waveguide will be higher than that at the top (Supplementary Fig. 3f). From Supplementary Fig. 3d it can be seen that the FoM of the TM strip waveguide plateaus when strip width is larger 400 nm; the cross sectional electric field distribution of a TM strip waveguide with width of 425 nm is indicated in Supplementary Fig. 3f.Comparing the FoM of the slot waveguides (Fig. 2b of the main text) against that of the TE and TM slot waveguides (Supplementary Fig. 3d), it can be seen that significantly higher FoM and thus surface sensitivity can be realized for slot waveguides. To that effect, slot waveguides are implemented for the Pb^{2+} photonic sensor presented in this work.

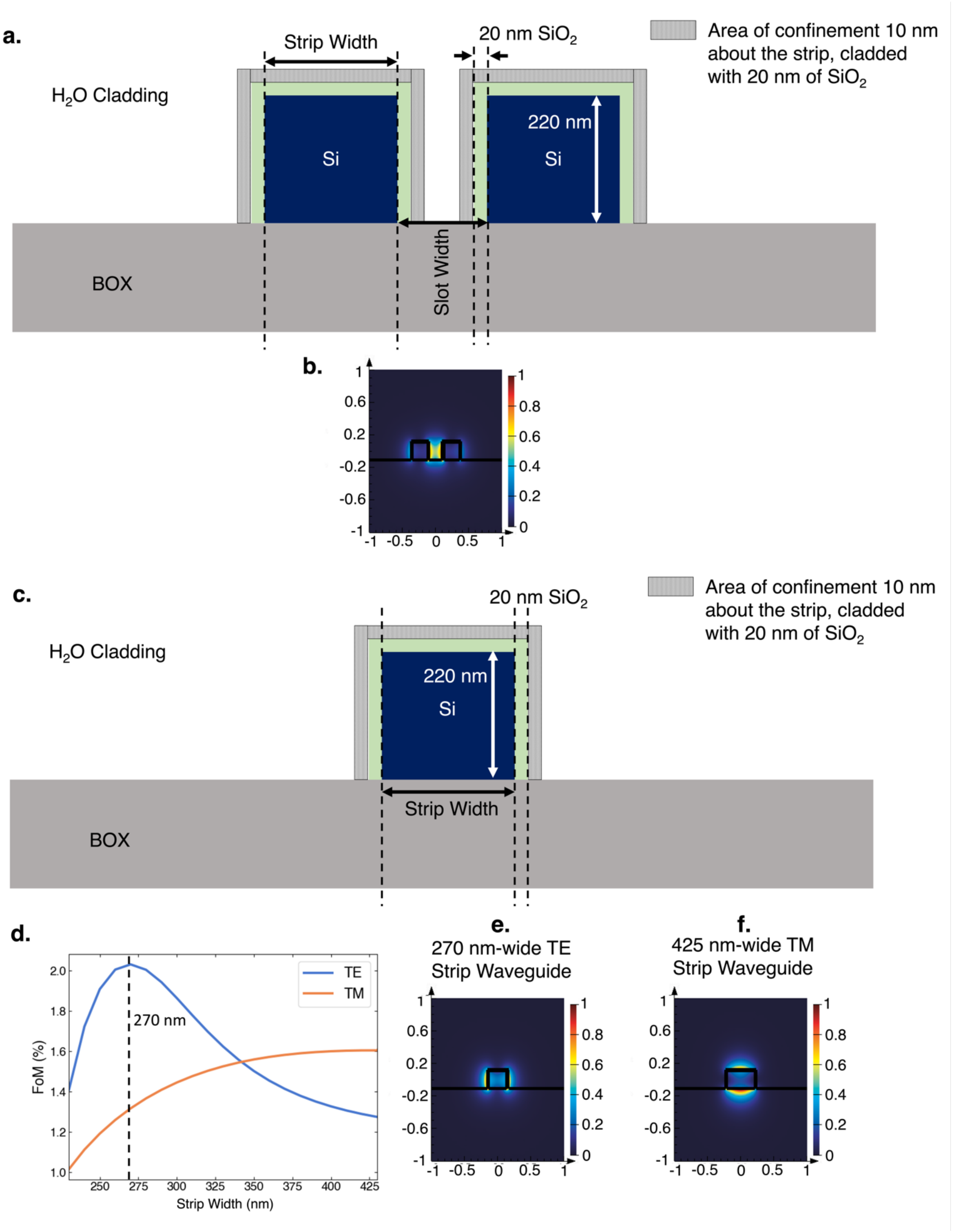

Supplementary Fig. 3. Definition of FoM for the optimization of waveguide surface sensitivity. The height of the waveguide layer is 220 nm with a 20 nm SiO_2 layer surrounding the waveguide, deposited via ALD. **a,** Cross-sectional schematic of the slot waveguide with definition of slot and strip width illustrated; the area of confinement is illustrated. **b,** An instance of electric field distribution pertaining to a slot waveguide with slot and strip widths of 240 nm. **c,** Cross-sectional schematic of the strip waveguide(TE/TM) with definition of strip width

illustrated; the area of confinement is illustrated. **d,** Sensor surface sensing FoM for strip waveguides (TE and TM) as a function of strip width. Instances of electric field distribution pertaining to **e,** TE strip waveguide with width of 270 nm, and **f,** TM strip waveguide with width of 425 nm.

Supplementary Note 5: Derivation of Equation 3 in the main text, relating designed loss (water absorption) with optimal splitter power ratios

Condition 1 is illustrated in Supplementary Fig. 4a. A lightwave ($I = |\mathrm{E}^2|$) is injected into an asymmetrical splitter, with two power splitting ratios; $\mathrm{S}_1, \mathrm{S}_1'$ (input splitter) and $\mathrm{S}_2, \mathrm{S}_2'$ (output splitter) where, $\mathrm{S}_1 = \mathrm{S}_2$ and $\mathrm{S}_1' = \mathrm{S}_2'$, $\mathrm{S}_{1/2}' \neq 0.5$. Assuming the splitters are lossless, energy conservation dictates that $\mathrm{S}_{1/2} = 1 - \mathrm{S}_{1/2}'$. After the input splitter, the field is separated into the sensing and reference arm, yielding E_1 and E_2 respectively. These two terms are related to the electric field at the input of the splitter (E) through the splitting ratio such that $\mathrm{E}_1 = E\sqrt{\mathrm{S}_1}$ and $\mathrm{E}_2 = E\sqrt{\mathrm{S}_1{}'}$. As the light power propagates through the sensing arm, it will accumulate attenuation from water absorption which we define as $e^{-\alpha L}$, with L being the sensing arm length and α is the attenuation constant, as compared to the reference arm. We can express the phase difference between the two MZI arms as $e^{-j\Delta\phi}$.

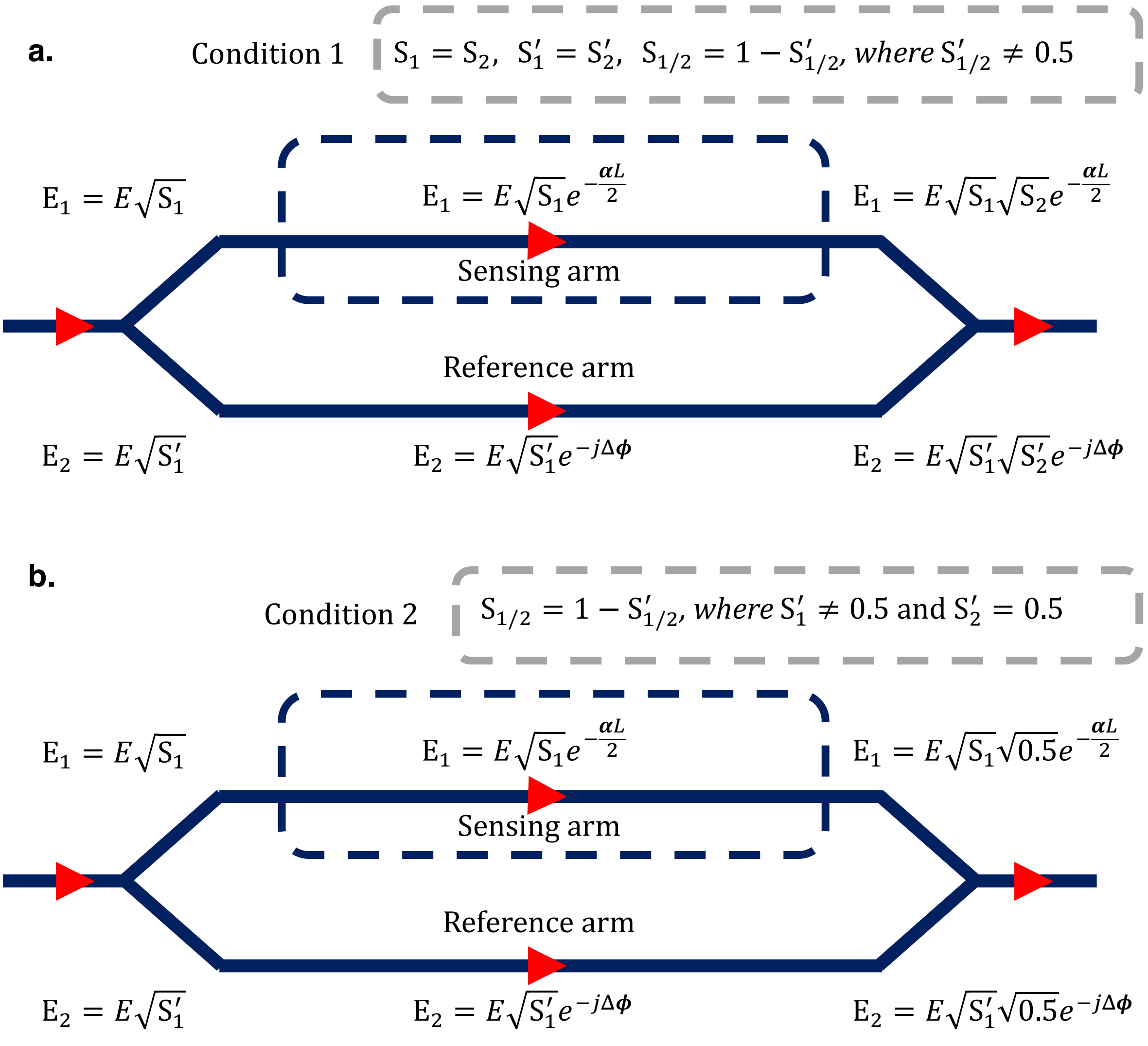

Supplementary Fig 4. Architectures of two MZI-based sensor designs with a, Condition 1, $S_1 = S_2, S_1' = S_2', S_{1/2} = 1 - S_{1/2}'$, where $S_{1/2}' \neq 0.5$, and **b,** Condition 2, $S_{1/2} = 1 - S_{1/2}', where\ S_1' \neq 0.5$, and $S_2' = 0.5$. According to Fig. 2c of the main text, it can be seen that the architecture in Supplementary Fig. 4a, condition 1 imposes lower requirements on splitter asymmetry as compared to Supplementary Fig. 4b, condition 2.

Lastly, as the two fields recombine at the output splitter, the total output intensity will be of the form:

$$I_{out} = E^2|\sqrt{S_1}\sqrt{S_2}e^{-\frac{\alpha L}{2}} + \sqrt{S_1'}\sqrt{S_2'}e^{-j\Delta\phi}|^2 \quad (S5.1)$$

In order to maximize sensor visibility, $I_{out} = 0$ during destructive interference condition. As a result, (S5.1) can be reduced to the following form,

$$\sqrt{S_1}\sqrt{S_2}e^{-\frac{\alpha L}{2}} = \sqrt{S_1{}'}\sqrt{S_2'} \qquad (S5.2)$$

An alternative MZI architecture (condition 2) is also illustrated in Supplementary Fig. 4b. In this design, an arbitrary and 3-dB splitter are used at the input and output respectively; $S_1' \neq 0.5, S_2' = 0.5$. Similar to condition 1, the splitters are also assumed to be lossless where the energy is conserved, $S_{1/2} = 1 - S_{1/2}'$. The output of the MZI can then be defined as the following.

$$I_{out} = E^2|\sqrt{S_1}\sqrt{0.5}e^{-\frac{\alpha L}{2}} + \sqrt{S_1{}'}\sqrt{0.5}e^{-j\Delta\phi}|^2 \quad (S5.3)$$

Similarly, to maximize sensor visibility, we set $I_{out} = 0$. As such, Equation (S5.3) can be reduced to.

$$\sqrt{S_1}e^{-\frac{\alpha L}{2}} = \sqrt{S_1'} \qquad (S5.4)$$

Via Equation (S5.2) and (S5.4), we determine the optimal splitting ratios of the arbitrary splitters as a function of designed losses for the MZI architectures in Supplementary Fig. 4a-b; designed loss from water absorption is assumed to be the only source of optical loss. From Fig. 2c of the main text, it can be seen that the MZI structure illustrated in Supplementary Fig. 4a (condition 1) reduces the asymmetrical requirement in power splitting as compared to Supplementary Fig. 4b (condition 2), which significantly alleviates requirement on fabrication ratios. Accurate fabrication of highly asymmetrical power splitters is challenging; small variations in splitter dimensions will result in significant changes from the intended design.

Supplementary Note 6: EDX analysis prior and after functionalization, and after Pb^{2+} exposure and analyte flush and dry (N_2)

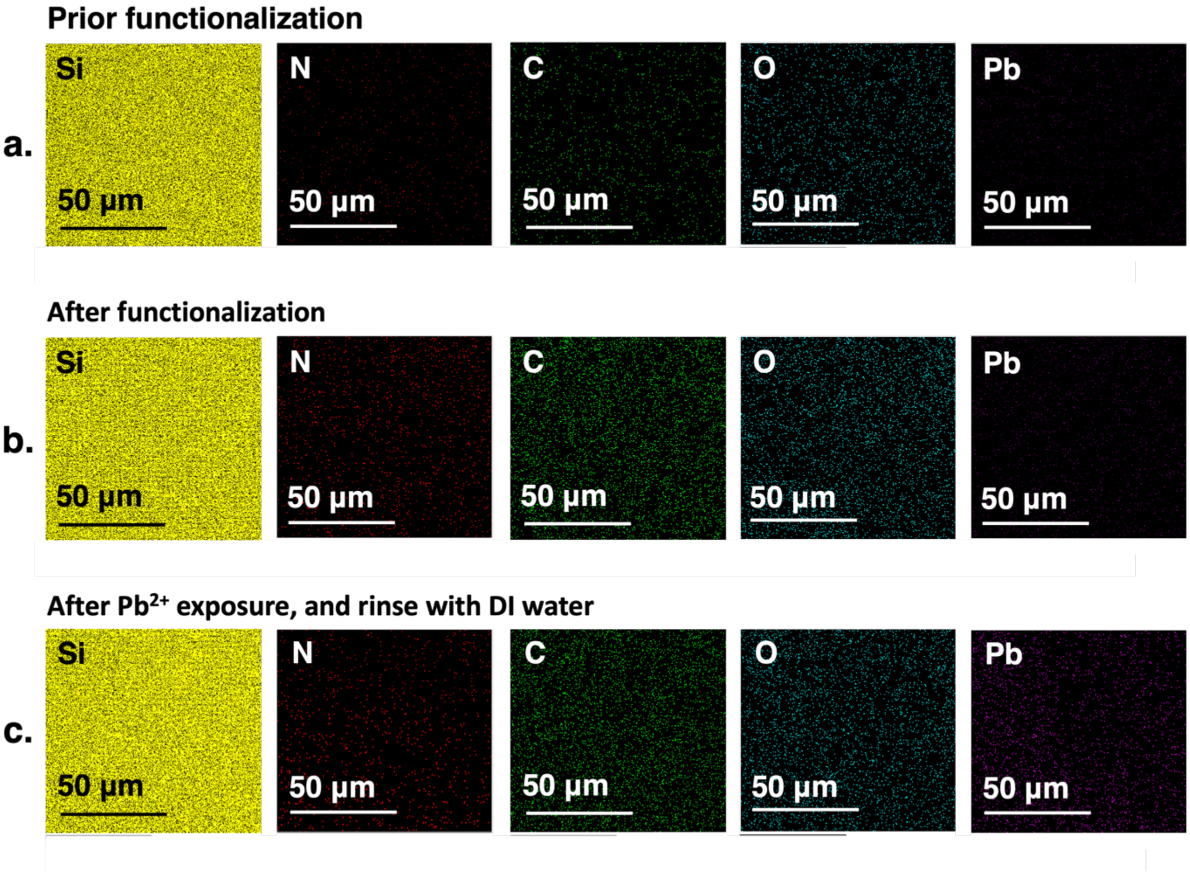

Supplementary Fig. 5 EDX analysis of the photonic chips pertaining to Si, N, C, O, and Pb elemental composition a, prior functionalization, **b,** after functionalization, and **c,** after Pb^{2+} in DI water exposure (120 s), analyte flush and dry (N_2). In Supplementary Fig. 5c, the pH of the Pb^{2+} analyte is 6.8 (see Methods).

In addition to the XPS results shown in the Fig. 3b-d, 4I of the main text, an EDX analysis was carried out to further validate the elemental composition of Si, N, C, O and Pb on the surface of the photonic chips. The analysis of N, C, and O indicates the viability of the Fischer esterification protocol that was developed in this work[39]. By comparing Supplementary Fig. 5a with Supplementary Fig. 5b or Supplementary Fig. 5c, an increase in N and C elemental composition can be observed. This is in line with the conclusion derived from the N *1S* and C *1S* regions of the XPS spectrums shown in Fig. 3b-c of the main text. We were not able to observe a change in O elemental composition in the EDX analysis. This is due to the fact that the O signal is attributed from the functional layer, as well as the 20 nm SiO_2 that was deposited

on the slot waveguides prior functionalization. As the 20 nm SiO_2 layer is the primary contributor to the O signal in Supplementary Fig. 5[40], a clear change in signal intensity cannot be seen. To further validate the capacity of the functional layer to bind with Pb^{2+} ions, the functional layer is exposed to Pb^{2+} (DI water, pH = 6.8) for 120 s, followed by analyte flush with DI water and drying (N_2). By comparing the rightmost column of Supplementary Fig. 5b and c, the appearance of Pb via the binding capabilities of the crown ether functional layer can be clearly observed.

Supplementary Note 7: Detection of Sn^{2+} through XPS analysis after functionalization

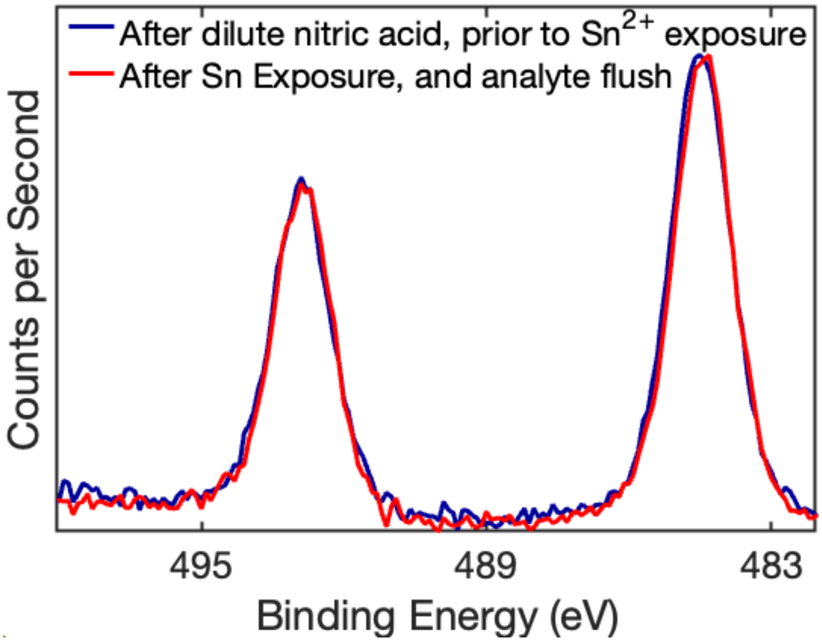

Supplementary Fig. 6 Sn^{2+} XPS spectrum measured after dilute HNO_3 purification (Fig. 3a of the main text) prior to Sn^{2+} exposure, and after Sn^{2+} exposure (DI water, pH = 6.8) with analyte flush and dry (N_2).

Due to the application of H_2O as a green solvent for the developed reaction in this work, Brønsted acid catalyst such as H_2SO_4 is incompatible, in view of its drastic decrease in catalytic activity, in the presence of H_2O[41]. To that effect, the Lewis acid catalyst, $SnCl_2$ is utilized, which has a catalytic activity that is more resilient to the presence of H_2O[42,43]. While Fischer esterification is favored when H_2O is removed as the reaction proceeds (dehydrative esterification), for the reaction disclosed in this work, Sn is embedded within the SiO_2 substrate,

forming a heterogeneous catalyst. Thereby, improved catalytic activity[44] that favors esterification in the presence of H_2O is achieved[45]. Evidence of successful Fischer esterification is indicated by the XPS N *1S*, C *1S*, and O *1S* data in Fig. 3b-d of the main text respectively, and the EDX analysis in Supplementary Fig. 5. Furthermore, Supplementary Fig. 6 shows the XPS results of the functional layer after dilute nitric acid purification (process in Fig. 3a of the main text), prior to Sn^{2+} exposure. It can be seen that presence of Sn cannot be eliminated via the purification step. We note that heterogeneous catalyst displays improved catalytic activity that favors esterification, even in the presence of H_2O. Supplementary Fig. 6 also shows the XPS measurement of the functional layer after Sn^{2+} exposure at 15 ppb, followed by analyte flush and drying. As the DBTDA crown ethers that undergoes amine conjugation subsequently do not bind to Sn^{2+} ions, it can be seen that the XPS spectrum is similar to that prior Sn^{2+} exposure. The subtraction of the narrow scan XPS spectrum before ion interaction to that after ion interaction, refers to the normalized XPS spectrum, as shown in Fig. 4j of the main text.

Supplementary Note 8: Details on chip fabrication

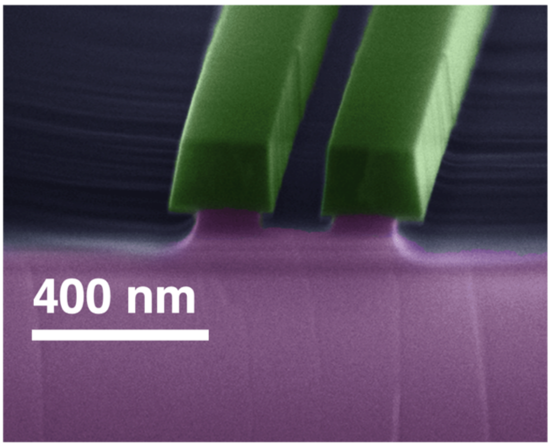

Supplementary Fig. 7 The SEM image (false colour) of fabricated slot waveguide, with strip and slot widths of 240 nm.

The fabrication of the sensor chips starts from commercially available 200 mm silicon on insulator wafers with 3 µm thick buried oxide and 220 nm thick device layer. First, the wafers are cleaned using a heated acetone bath kept at 55°C and rinsed in methanol, isopropanol and DI water. Then, an adhesion promoter (Surpass 4000) and electron-sensitive resist (ma-N 2403) are spin-coated onto the wafer and baked for 2 minutes at 90°C. The thickness of the E-beam resist after spin coating is ~300 nm (achieved at spin speeds of 3000 rounds per minute). A discharging layer (Espacer-300Z from Showa Denko Inc.) is applied to minimize charging effects. The wafer is patterned using E-beam lithography (ELS-HS50 from STS-Elionix) with a 50 kV accelerating voltage and a beam current of 5 nA. After development in RD6 (Futurexx Inc.) for 80s and rinsing in DI water, the waveguides are etched using ICP-RIE (RIE-230iP from Samco Inc.) with a gas chemisty of CF_4 and Ar at a pressure of 1 Pa, ICP Power of 300 W and 100 W of bias Power. The wafer is then ashed in O_2 plasma to strip away any remaining E-beam resist and to remove residual fluoropolymer formed during the etching process and thoroughly cleaned in Piranha solution, followed by a DI water rinse. Subsequently, the wafer was cladded with 2 µm of SiO_2 deposited at 350°C vie PECVD (Samco PD-220NL from Samco Inc). The wafer was then baked at 115 °C and silanized in an oven (TA Series from

Yield Engineering Systems Inc.) to increase adhesion promotion of photoresist. Then, a thin (~1 μm) AZ 3312 photoresist layer is spin-coated and softbaked at 110°C for 60s. The sensing trench pattern is exposed into the resist using a maskless aligner (MLA-150 from Heidelberg Instruments Mikrotechnik GmbH) with a laser source centered at 405 nm. The resist was then post-exposure baked at 110 °C for 60 s and developed using AZ 726 MIF developer (Microchemicals GmbH) for 60 s. A diluted buffered oxide etchant solution was then used to open the sensing trenches, exposing the waveguides in the sensing arm. To avoid overetching, which could suspend the waveguides and generally change the structural cross-section from the intended design, the etching depth was monitored during the etching process using both profilometry (Dektak-XT from Bruker Corporation) and reflectometry (F50-UVX from Filmetrics Inc.). Once a total etch depth of 2 μm is reached, the resist was stripped in oxygen plasma (e3511 wafer asher from ESI Inc.), followed by thorough cleaning in acetone, isopropanol and DI water to prepare the wafer for the crown ether functionalization step.

Supplementary Note 9: Measured optical spectrum of the photonic sensor when the sensing region is exposed to air

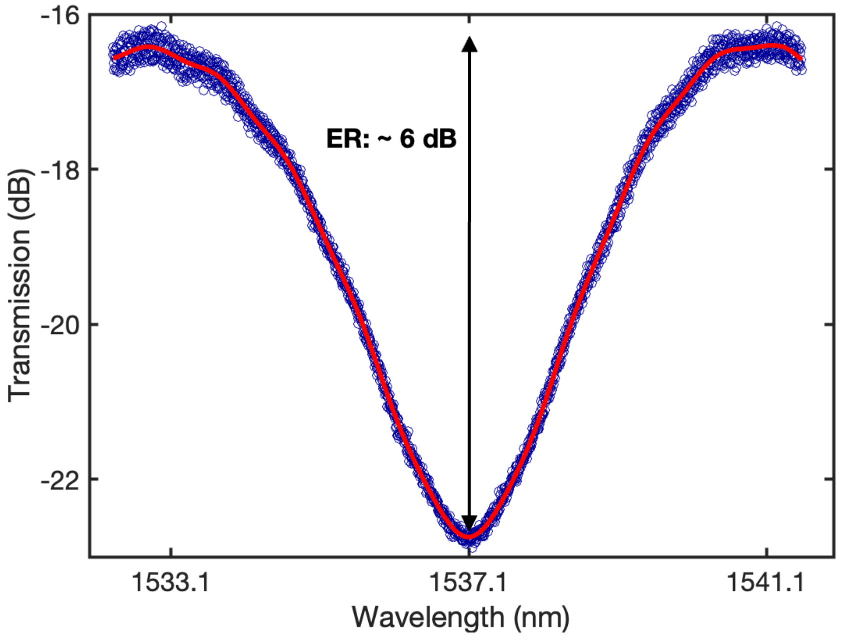

Supplementary Fig. 8. Measured optical spectrum of the photonic sensor when the sensing region of the photonic sensor is exposed to air. Poor visibility, as implied from the interferometric spectrum extinction ratio is observed, which is resultant when the designed losses are non-optimal to the designed asymmetrical splitting ratios.

Supplementary Note 10: Mathematical relationship between sensor resonant shift and exposed Pb^{2+} concentrations

In Fig. 5b of the main text, Pb^{2+} concentrations of 5, 25, 125, 625, 2625 12625, 62625, 262625 ppb are measured through a cumulative testing process (see Methods), across 6 independent sensors each ($n = 6$). The average resonant wavelength shift at each concentration was taken, and fitted to the following equation:

$$\text{Resonance shift } (\lambda_s - \lambda_0)_{\text{average}} = \frac{a}{1 + \exp(-b \times (\text{conc.} - c))} \quad (8.1)$$

where $\mathbf{conc.}$ is the reference concentration of exposed Pb^{2+} ions in the log-scale. $\boldsymbol{a, b, c}$ are the fitting parameters. The shape of the curve is characteristic of (i) absorption isotherms[46,47], attributed by the binding of Pb^{2+} ions to the crown ether functional layer, and (ii) light-matter interaction between the waveguide mode and the functional layer with Pb^{2+} binded. This curve is found to accurately model the sensor resonant shift, and saturation of the binding sites on the crown ether decorated silicon photonic sensor as the concentration of Pb^{2+} increases.

Supplementary Note 11: Definition of crown ether decorated photonic sensor lower and upper detection limit

The following defines the lower and upper detection limits of the sensor. The standard deviation of the sensor noise (σ_n) is measured to be 0.014 nm, obtained from ten repeated resonant wavelength measurements of a photonic sensor, exposed to DI water. This accounts for the inherent sensor resonance drift due to environmental thermal variations as well as the accuracy of the measurement setup in determining the resonant wavelength. In view of the significant detection dynamic range, the lower and upper detection limit of the sensor needs to be assessed separately by absolute and fractional accuracy of detection respectively. The lower limit of detection of Pb^{2+} is found to be the at 0.882 ppb, where the corresponding value of (λ_s-λ_0) = 0.042 nm is three times the standard deviation ($3\sigma_n$) of the noise[8]. On the other hand, the upper detection limit is determined by the assessing the difference in (λ_s-λ_0), ±10 % about the average resonance shift $(\lambda_s\text{-}\lambda_0)_{average}$[48] (Supplementary Note 10) of the highest tested concentration, that is larger than $3\sigma_n$[8]. This criterion will enable the confident quantification of Pb^{2+} concentration levels within the range. To that effect, the upper limit is determined to be 62000 ppb, where the difference in (λ_s-λ_0) about $(\lambda_s\text{-}\lambda_0)_{mean}$ between concentrations at ±10 % of 62000 ppb is 0.068 nm; the value of $3\sigma_n$ = 0.042 nm.

Supplementary Note 12: Sensor Reusability

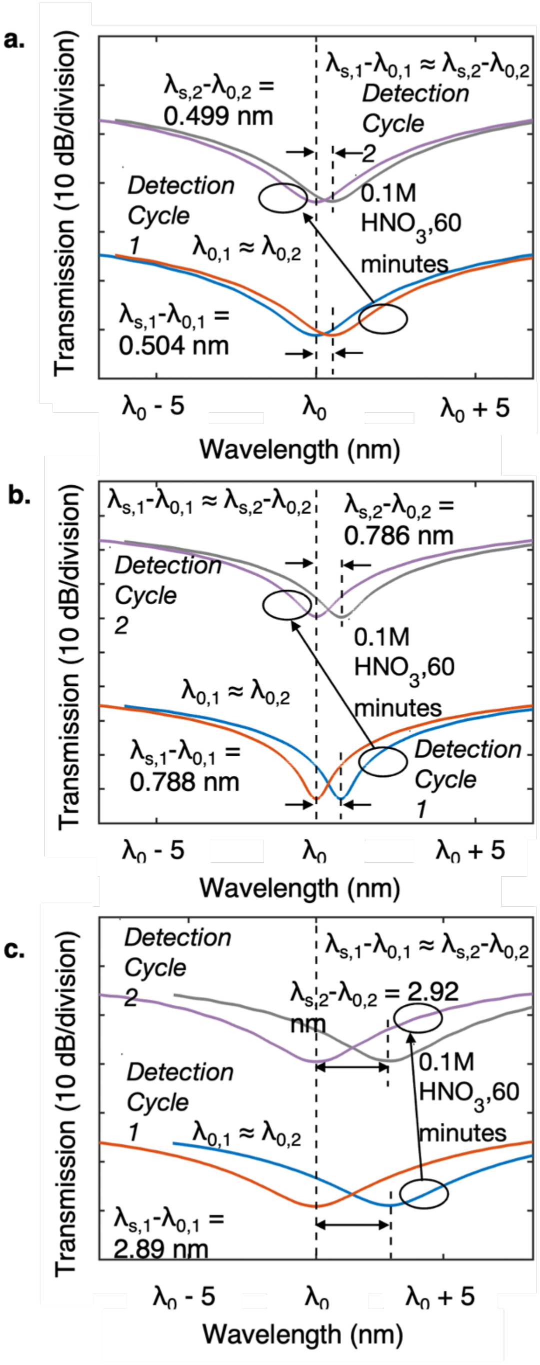

Supplementary Fig. 9 Demonstration of sensor reusability. The optical fringe minima measured in DI water to determine $\lambda_{0,1}$, $\lambda_{0,2}$ and after analyte flush to determine $\lambda_{s,1}$, $\lambda_{s,2}$ performed over two detection cycles at Pb^{2+} concentration of **a,** 15 ppb in DI water (pH = 6.8) **b,** 23 ppb in sea water (pH = 7.79) **c,** 80 ppb in DI water (pH = 6.8). The optical fringe minima for $\lambda_{0,1}$ and $\lambda_{s,1}$ in a-c corresponds to the spectrum measured at reference and after analyte flush in Fig. 6c of the main text, Supplementary Fig. 11c, and 5e of the main text respectively. The optical fringe minima for $\lambda_{0,2}$ is measured after sensor regeneration through exposure to 0.1 M of HNO_3 for 60 minutes.

The optical fringe minima for $\lambda_{s,2}$ is measured after 120 s of Pb^{2+} exposure for each of the abovementioned tested analyte and followed by analyte flush.

The binding of Pb^{2+} to DBTDA crown ethers is facilitated through coordinate covalent bonds, formed between the electron deficient Pb^{2+}, and the electron rich oxygen and nitrogen atoms on the crown ether. This results in the formation of a stable complex in solution[49,50].

This binding process is optimal when the oxygen and nitrogen within the crown ether is unprotonated. It is of note that the acidification of the environment surrounding the crown ether promotes the protonation of oxygen and nitrogen atoms on the crown ether, via the addition of H^+ ions[51]. This leads to a decrease in the availability of active sites to form coordination bonds with Pb^{2+}. Furthermore, the introduction of positive charges would lead to the repelling of positively-charged Pb^{2+}. The above results in a reduction in the binding affinity between Pb^{2+} and the DBTDA crown ether. As such, Pb^{2+} which are bound to the crown ether will be released as free ions[49].

Based on the above principle, the reusability of the crown ether decorated silicon photonic Pb^{2+} sensor platform is assessed via acidification, to release Pb^{2+} ions from the DBTDA crown ethers. 0.1 M of dilute nitric acid (HNO_3) is applied for 60 minutes to the three photonic sensors previously exposed to Pb^{2+} reference concentrations of 15, 80, 23 ppb (Fig. 6c, 5e of the main text, and Supplementary Fig. 11c respectively). To demonstrate sensor reusability, Supplementary Fig. 9a-c shows the corresponding optical spectra for two Pb^{2+} detection cycles with sensor regeneration carried out after the first detection step ($\lambda_{s,1}$). The elucidated protocol is as below: 1.) The sensors are exposed to DI water to obtain the first reference resonant wavelength ($\lambda_{0,1}$), and flushed afterwards from the microfluidic chamber. 2.) The three separate sensors are then exposed to 15, 80, 23 ppb of Pb^{2+} respectively for 120 s. The detection of 15, and 80 ppb of Pb^{2+} were performed in DI water, with pH of 6.8. On the other hand, the detection of 23 ppb of Pb^{2+} were performed in sea water, with pH of 7.79. 3.) Following, analyte flush is implemented where the ($\lambda_{s,1}$) is obtained; the definition of analyte flush is defined in the main

text. The sensor inferred concentrations are obtained via ($\lambda_{s,1}$- $\lambda_{0,1}$), and the calibration curve in Fig. 5b of the main text. 4.) With the conclusion of the first detection step, where the corresponding spectrum is shown in Fig. 6c, 5e of the main text, and Supplementary Fig. 11c, the sensors are regenerated through exposure to 0.1 M of HNO_3 for 60 minutes. This releases the Pb^{2+} ions from the DBTDA crown ether. Following, the free ions are flushed out of the microfluidic chamber via DI water. 5.) After the regeneration of the sensors, Steps 1 to 3 are then repeated again to obtain the second reference ($\lambda_{0,2}$) and detection wavelength ($\lambda_{s,2}$). From Supplementary Fig. 9a-c, $\lambda_{0,2} = \lambda_{0,1}$. This indicates that the acidification process releases Pb^{2+} ions from the DBTDA crown ethers, resulting in the subsequent drop in the material index of the crown ether functional layer where $\lambda_{s,1}$ reverts back to $\lambda_{0,1}$. From Supplementary Fig. 9a-c, it can be seen that $\lambda_{s,1}$-$\lambda_{0,1} \approx \lambda_{s,2}$-$\lambda_{0,2}$, implying a close match in sensor inferred concentrations between the two detection cycles. To further validate the protocol, the reusability of the sensors is subjected to the regeneration process through six separate sensors (n = 6) at each of the concentrations. The mean inferred concentration ($Conc._{mean}$) standard deviation ($\sigma_{conc.}$) are presented in Supplementary Table 2, closely approximating ground truth values. Therefore, it can be concluded that the acidification approach mentioned will enable photonic sensor regeneration and reuse.

**Reference Pb^{2+} concentration and analyte pH**	**Inferred Conc.$_{mean}$/ $\sigma_{conc.}$ (*n* = *6*) from the first detection**	**Inferred Conc.$_{mean}$/ $\sigma_{conc.}$ (*n* = *6*) from the second detection**
15 ppb in DI water, pH = 6.8	15.5 ppb/ 0.7 ppb/ 3.3 %	15.4 ppb/ 0.6 ppb/ 2.7 %
23 ppb in synthetically doped sea water; concentration of Pb^{2+} increased by 15 ppb. pH = 7.79	22.8 ppb/ 0.7 ppb/ 0.9 %	22.6 ppb/ 0.8 ppb/ 1.7 %
80 ppb in DI water, pH = 6.8	80.9 ppb/ 2.0 ppb/ 1.1 %	82.4 ppb/ 2.9 ppb/ 3.0 %

Supplementary Table 2 Demonstration of sensor detection accuracy and reusability. Pb^{2+} concentrations are 15 ppb in DI water (pH = 6.8), 23 ppb in sea water (pH = 7.79) and 80 ppb in DI water (pH = 6.8) over two detection cycles. Conc.$_{mean}$ refers to mean sensor inferred concentrations. $\sigma_{conc.}$ refers to standard deviation of the sensor inferred concentrations. Err. refers to the variation in Conc.$_{mean}$ from the reference concentrations. To investigate the repeatability of the protocol, six separate sensors (*n* = *6*) are tested at each concentration. The reference Pb^{2+} concentrations are verified via ICP-MS.

Supplementary Note 13: Pb^{2+} ion photonic sensor selectivity test against Na^{+}, Mg^{+}, Cu^{+}, Sn^{+2}, Ca^{+2}, Fe^{2+}

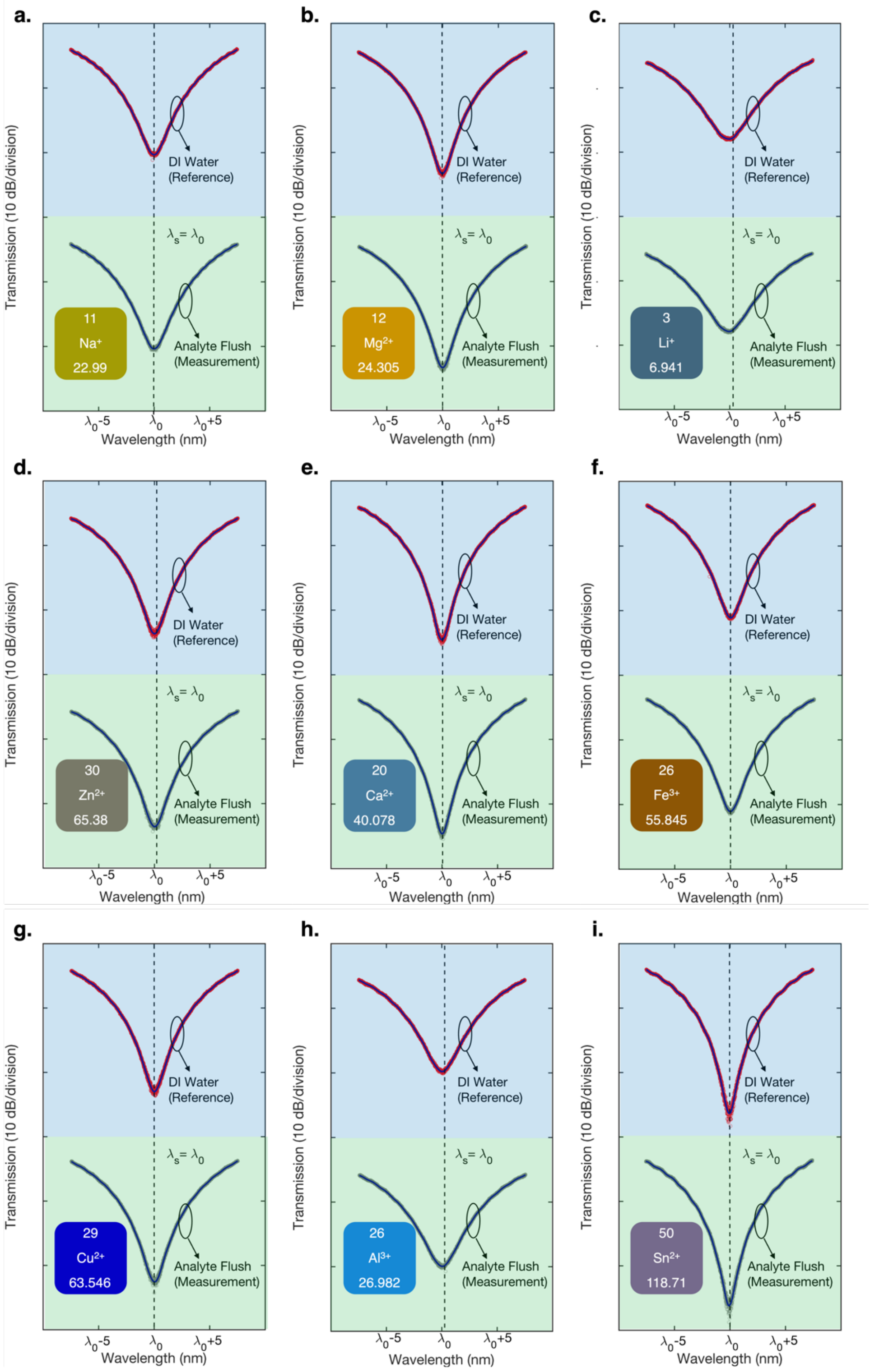

Supplementary Fig. 10 Selectivity performance of the Pb^{+2} ion sensor against a, Na^{+}, **b,** Mg^{2+}, **c,** Li^{+} **d,** Zn^{2+}, **e,** Ca^{2+}, **f,** Fe^{3+}, **g,** Cu^{2+}, **h,** Al^{3+}, **i,** Sn^{2+} at 15 ppb reference concentrations in DI water where no shifts in the interferometric spectra indicative of ion binding is observed across all the tested ions. The pH of the analyte is maintained at 6.8 (see Methods).

Supplementary Note 14: ICP-MS elemental analysis of environmentally related scenarios; tap lake and sea water

Elemental distribution in tap water	Ground truth concentration values
Silver (Ag)	< 20 ppb
Aluminum (Al)	40 ppb
Arsenic (As)	< 10 ppb
Boron (B)	150 ppb
Barium (Ba)	< 20 ppb
Beryllium (Be)	< 10 ppb
Calcium (Ca)	33380 ppb
Cadmium (Cd)	< 2 ppb
Cobalt (Co)	< 20 ppb
Chromium (Cr)	< 20 ppb
Copper (Cu)	50 ppb
Iron (Fe)	40 ppb
Mercury (Hg)	< 1 ppb
Potassium (K)	4800 ppb
Lithium (Li)	< 20 ppb
Magnesium (Mg)	2300 ppb
Manganese (Mn)	< 20 ppb
Molybdenum (Mo)	< 20 ppb
Sodium (Na)	32410 ppb
Nickel (Ni)	< 20 ppb
Phosphorous (P)	< 20 ppb

Lead (Pb)	2 ppb
Palladium (Pd)	< 20 ppb
Sulphur (S)	17520 ppb
Antimony (Sb)	20 ppb
Selenium (Se)	20 ppb
Silicon (Si)	1300 ppb
Tin (Sn)	< 20 ppb
Strontium (Sr)	50 ppb
Titanium (Ti)	< 20 ppb
Thallium (Tl)	< 20 ppb
Vanadium (V)	< 20 ppb
Zinc (Z)	20 ppb
Aurum (Au)	< 20 ppb
Bismuth (Bi)	< 20 ppb
Platinum (Pt)	< 20 ppb

Supplementary Table 3 ICP-MS analysis of tap water, indicating the constituent elemental distribution.

Elemental distribution in lake water	Ground truth concentration values
Silver (Ag)	< 20 ppb
Aluminum (Al)	640 ppb
Arsenic (As)	< 10 ppb
Boron (B)	20 ppb
Barium (Ba)	20 ppb
Beryllium (Be)	< 20 ppb
Calcium (Ca)	22170 ppb

Cadmium (Cd)	< 2 ppb
Cobalt (Co)	< 20 ppb
Chromium (Cr)	< 20 ppb
Copper (Cu)	< 20 ppb
Iron (Fe)	230 ppb
Mercury (Hg)	< 1 ppb
Potassium (K)	2600 ppb
Lithium (Li)	< 20 ppb
Magnesium (Mg)	1400 ppb
Manganese (Mn)	20 ppb
Molybdenum (Mo)	< 20 ppb
Sodium (Na)	10050 ppb
Nickel (Ni)	< 20 ppb
Phosphorous (P)	20 ppb
Lead (Pb)	5 ppb
Palladium (Pd)	< 20 ppb
Sulphur (S)	8890 ppb
Antimony (Sb)	30 ppb
Selenium (Se)	< 10 ppb
Silicon (Si)	3470 ppb
Tin (Sn)	< 20 ppb
Strontium (Sr)	60 ppb
Titanium (Ti)	< 20 ppb
Thallium (Tl)	20 ppb

Vanadium (V)	< 20 ppb
Zinc (Z)	< 20 ppb
Aurum (Au)	< 20 ppb
Bismuth (Bi)	< 20 ppb
Platinum (Pt)	< 20 ppb

Supplementary Table 4 ICP-MS analysis of lake water, indicating the constituent elemental distribution.

Elemental distribution in sea water	Ground truth concentration values
Silver (Ag)	< 20 ppb
Aluminum (Al)	160 ppb
Arsenic (As)	< 10 ppb
Boron (B)	2030 ppb
Barium (Ba)	160 ppb
Beryllium (Be)	< 20 ppb
Calcium (Ca)	287700 ppb
Cadmium (Cd)	< 2 ppb
Cobalt (Co)	< 20 ppb
Chromium (Cr)	< 20 ppb
Copper (Cu)	< 20 ppb
Iron (Fe)	30 ppb
Mercury (Hg)	< 1 ppb
Potassium (K)	282300 ppb
Lithium (Li)	170 ppb
Magnesium (Mg)	876500 ppb
Manganese (Mn)	< 20 ppb

Molybdenum (Mo)	< 20 ppb
Sodium (Na)	6922000 ppb
Nickel (Ni)	< 20 ppb
Phosphorous (P)	40 ppb
Lead (Pb)	8 ppb
Palladium (Pd)	< 20 ppb
Sulphur (S)	573600 ppb
Antimony (Sb)	40 ppb
Selenium (Se)	< 10 ppb
Silicon (Si)	1.170 ppb
Tin (Sn)	< 20 ppb
Strontium (Sr)	4870 ppb
Titanium (Ti)	< 20 ppb
Thallium (Tl)	< 20 ppb
Vanadium (V)	130 ppb
Zinc (Z)	< 20 ppb
Aurum (Au)	< 20 ppb
Bismuth (Bi)	< 20 ppb
Platinum (Pt)	740 ppb

Supplementary Table 5 ICP-MS analysis of sea water, indicating the constituent elemental distribution.

Supplementary Note 15: Pb^{2+} photonic sensor performance in tap, lake and sea water, where the Pb^{2+} concentrations are synthetically increased by 15 ppb verified by ICP-MS

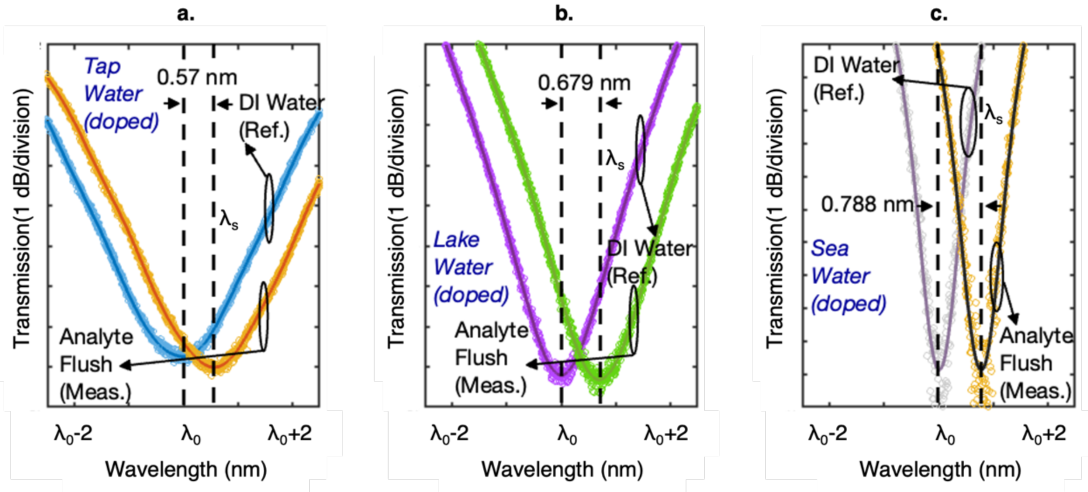

Supplementary Fig. 11 Pb^{2+} sensor deployment in environmentally related situations where the concentration of tap, lake and sea water are synthetically increased by 15 ppb to a, 17, **b,** 20, **c,** 23 ppb. The pH of the synthetically doped tap, lake and sea water are 8.26, 7.91, and 7.79 respectively.

References

1. Zhan, S. *et al.* Label-free fluorescent sensor for lead ion detection based on lead(II)-stabilized G-quadruplex formation. *Anal. Biochem.* **462**, 19–25 (2014).
2. Du, X. *et al.* A Fluorescence Sensor for Pb2+ Detection Based on Liquid Crystals and Aggregation-Induced Emission Luminogens. *ACS Appl. Mater. Interfaces* **13**, 22361–22367 (2021).
3. Marbella, L., Serli-Mitasev, B. & Basu, P. Development of a Fluorescent Pb2+ Sensor. *Angew. Chem. Int. Ed.* **48**, 3996–3998 (2009).
4. Niu, X. *et al.* A “turn-on” fluorescence sensor for Pb2+ detection based on graphene quantum dots and gold nanoparticles. *Sens. Actuators B Chem.* **255**, 1577–1581 (2018).
5. Munir, A. *et al.* Selective and simultaneous detection of Zn2+, Cd2+, Pb2+, Cu2+, Hg2+ and Sr2+ using surfactant modified electrochemical sensors. *Electrochim. Acta* **323**, 134592 (2019).
6. Zhang, T. *et al.* Detection of trace Cd2+, Pb2+ and Cu2+ ions via porous activated carbon supported palladium nanoparticles modified electrodes using SWASV. *Mater. Chem. Phys.* **225**, 433–442 (2019).
7. Hwang, J.-H. *et al.* Improving Electrochemical Pb2+ Detection Using a Vertically Aligned 2D MoS2 Nanofilm. *Anal. Chem.* **91**, 11770–11777 (2019).
8. Wang, Y. *et al.* PPy-Functionalized NiFe2O4 Nanocomposites toward Highly Selective Pb2+ Electrochemical Sensing. *ACS Sustain. Chem. Eng.* **10**, 6082–6093 (2022).
9. Liu, J. & Lu, Y. Accelerated Color Change of Gold Nanoparticles Assembled by DNAzymes for Simple and Fast Colorimetric Pb2+ Detection. *J. Am. Chem. Soc.* **126**, 12298–12305 (2004).

10. Zhang, L. *et al.* A solid-state colorimetric fluorescence Pb2+-sensing scheme: mechanically-driven CsPbBr3 nanocrystallization in glass. *Nanoscale* **12**, 8801–8808 (2020).
11. Yap, S. H. K. *et al.* An Advanced Hand-Held Microfiber-Based Sensor for Ultrasensitive Lead Ion Detection. *ACS Sens.* **3**, 2506–2512 (2018).
12. Nyika, J., Onyari, E., Dinka, M. O. & Mishra, S. B. A Comparison of Reproducibility of Inductively Coupled Spectrometric Techniques in Soil Metal Analyses. *Air Soil Water Res.* **12**, 1178622119869002 (2019).
13. Douvris, C., Vaughan, T., Bussan, D., Bartzas, G. & Thomas, R. How ICP-OES changed the face of trace element analysis: Review of the global application landscape. *Sci. Total Environ.* **905**, 167242 (2023).
14. PalmSens4. https://www.palmsens.com/product/palmsens4/.
15. Ocean ST VIS Microspectrometer. https://www.oceaninsight.com/products/spectrometers/microspectrometer/st-series-spectrometer/ocean-st-vis-microspectrometer/.
16. Almost 1 million people die every year due to lead poisoning, with more children suffering long-term health effects. https://www.who.int/news/item/23-10-2022-almost-1-million-people-die-every-year-due-to-lead-poisoning--with-more-children-suffering-long-term-health-effects (2022).
17. Perry Gottesfeld. The Environmental And Health Impacts Of Lead Battery Recycling. https://wedocs.unep.org/bitstream/handle/20.500.11822/13943/1_ECOWAS%20lead%20background%202016.pdf.
18. Sun, C. *et al.* Single-chip microprocessor that communicates directly using light. *Nature* **528**, 534–538 (2015).
19. Sun, J., Timurdogan, E., Yaacobi, A., Hosseini, E. S. & Watts, M. R. Large-scale nanophotonic phased array. *Nature* **493**, 195–199 (2013).
20. Qiang, X. *et al.* Large-scale silicon quantum photonics implementing arbitrary two-qubit processing. *Nat. Photonics* **12**, 534–539 (2018).
21. Jurgens, B. C., Parkhurst, D. L. & Belitz, K. Assessing the Lead Solubility Potential of Untreated Groundwater of the United States. *Environ. Sci. Technol.* **53**, 3095–3103 (2019).
22. Understanding the Lead and Copper Rule. https://www.epa.gov/dwreginfo/lead-and-copperrule#:~:text=The%20rule%20replaced%20the%20previous,copper%20within%20the%20distribution%20system (2023).
23. Demir, F. & Derun, E. M. Modelling and optimization of gold mine tailings based geopolymer by using response surface method and its application in Pb2+ removal. *J. Clean. Prod.* **237**, 117766 (2019).
24. M.A. Barakat M.H. Ramadan, J. N. K. & Woodcock, H. L. Equilibrium and kinetics of Pb2+ adsorption from aqueous solution by dendrimer/titania composites. *Desalin. Water Treat.* **52**, 5869–5875 (2014).
25. Kita, D. M. *et al.* High-performance and scalable on-chip digital Fourier transform spectroscopy. *Nat. Commun.* **9**, 4405 (2018).
26. Sia, J. X. B. *et al.* Wafer-Scale Demonstration of Low-Loss (~0.43 dB/cm), High-Bandwidth (>38 GHz), Silicon Photonics Platform Operating at the C-Band. *IEEE Photonics J.* **14**, 1–9 (2022).
27. Fahrenkopf, N. M. *et al.* The AIM Photonics MPW: A Highly Accessible Cutting Edge Technology for Rapid Prototyping of Photonic Integrated Circuits. *IEEE J. Sel. Top. Quantum Electron.* **25**, 1–6 (2019).
28. Siew, S. Y. *et al.* Review of Silicon Photonics Technology and Platform Development. *J. Lightwave Technol.* **39**, 4374–4389 (2021).

29. Rahim, A. *et al.* Open-Access Silicon Photonics Platforms in Europe. *IEEE J. Sel. Top. Quantum Electron.* **25**, 1–18 (2019).
30. Sakai, T. & McCurdy, E. Agilent 7900 ICP-MS simplifies drinking water analysis. https://www.agilent.com/cs/library/applications/5991-4938EN.pdf.
31. Langevin, B. & Costedoat, M. Analysis of Heavy Metals in e-Liquids using the Agilent 5110 ICP-OES. https://www.agilent.com/cs/library/applications/5991-8676EN_e-liquids_icp-oes_application.pdf.
32. Almeida, V. R., Xu, Q., Barrios, C. A. & Lipson, M. Guiding and confining light in void nanostructure. *Opt. Lett.* **29**, 1209–1211 (2004).
33. Kita, D. M., Michon, J., Johnson, S. G. & Hu, J. Are slot and sub-wavelength grating waveguides better than strip waveguides for sensing? *Optica* **5**, 1046–1054 (2018).
34. Claes, T. *et al.* Label-Free Biosensing With a Slot-Waveguide-Based Ring Resonator in Silicon on Insulator. *IEEE Photonics J.* **1**, 197–204 (2009).
35. Palmer, R. *et al.* Low-Loss Silicon Strip-to-Slot Mode Converters. *IEEE Photonics J.* **5**, 2200409 (2013).
36. Liu, Q. *et al.* Highly sensitive Mach–Zehnder interferometer biosensor based on silicon nitride slot waveguide. *Sens. Actuators B Chem.* **188**, 681–688 (2013).
37. Tu, X. *et al.* Thermal independent Silicon-Nitride slot waveguide biosensor with high sensitivity. *Opt. Express* **20**, 2640–2648 (2012).
38. Ranno, L., Sia, J. X. B., Dao, K. P. & Hu, J. Multi-material heterogeneous integration on a 3-D photonic-CMOS platform. *Opt. Mater. Express* **13**, 2711–2725 (2023).
39. Khan, Z. *et al.* Current developments in esterification reaction: A review on process and parameters. *J. Ind. Eng. Chem.* **103**, 80–101 (2021).
40. Stefan Hüfner. *Photoelectron Spectroscopy Principles and Applications*. vol. 82 (Springer-Verlag Berlin Heidelberg New York, Berlin Heidelberg New York, 2003).
41. Liu, Y., Lotero, E. & Goodwin, J. G. Effect of water on sulfuric acid catalyzed esterification. *J. Mol. Catal. A Chem.* **245**, 132–140 (2006).
42. da Silva, M. J., Julio, A. A. & dos Santos, K. T. Sn(ii)-catalyzed β-citronellol esterification: a Brønsted acid-free process for synthesis of fragrances at room temperature. *Catal. Sci. Technol.* **5**, 1261–1266 (2015).
43. Casas, A., Ramos, M. J., Rodríguez, J. F. & Pérez, Á. Tin compounds as Lewis acid catalysts for esterification and transesterification of acid vegetable oils. *Fuel Process. Technol.* **106**, 321–325 (2013).
44. Xie, W., Wang, H. & Li, H. Silica-Supported Tin Oxides as Heterogeneous Acid Catalysts for Transesterification of Soybean Oil with Methanol. *Ind. Eng. Chem. Res.* **51**, 225–231 (2012).
45. Baek, H., Minakawa, M., Yamada, Y. M. A., Han, J. W. & Uozumi, Y. In-Water and Neat Batch and Continuous-Flow Direct Esterification and Transesterification by a Porous Polymeric Acid Catalyst. *Sci. Rep.* **6**, 25925 (2016).
46. Buttersack, C. Modeling of type IV and V sigmoidal adsorption isotherms. *Phys. Chem. Chem. Phys.* **21**, 5614–5626 (2019).
47. Altun, A. O., Bond, T., Pronk, W. & Park, H. G. Sensitive Detection of Competitive Molecular Adsorption by Surface-Enhanced Raman Spectroscopy. *Langmuir* **33**, 6999–7006 (2017).
48. T, W., H, J., A, S., P, R. & J, S. Guidance Document on the Estimation of LOD and LOQ for Measurements in the Field of Contaminants in Feed and Food. (2016) doi:10.2787/8931.
49. Lundgren, R. J. & Stradiotto, M. Key Concepts in Ligand Design. in *Ligand Design in Metal Chemistry* 1–14 (John Wiley & Sons, Ltd, 2016). doi:https://doi.org/10.1002/9781118839621.ch1.

50. Gupta, V. K., Jain, A. K. & Kumar, P. PVC-based membranes of N,N′-dibenzyl-1,4,10,13-tetraoxa-7,16-diazacyclooctadecane as Pb(II)-selective sensor. *Sens Actuators B Chem.* **120**, 259–265 (2006).
51. Golcs, Á., Vezse, P., Ádám, B. Á., Huszthy, P. & Tóth, T. Comparison in practical applications of crown ether sensor molecules containing an acridone or an acridine unit – a study on protonation and complex formation. *J. Incl. Phenom. Macrocycl. Chem.* **101**, 63–75 (2021).
