# Supplementary material for: Crown ether decorated silicon photonics for safeguarding against lead poisoning
Source: Nat Commun. 2024 May 14;15:3820. doi: 10.1038/s41467-024-47938-6 (PMC11094186; doi:10.1038/s41467-024-47938-6)
Supplement: Supplementary file 3 — Reporting Summary [file 41467_2024_47938_MOESM3_ESM.pdf]

nature portfolio

Corresponding author(s): Jia Xu Brian Sia

Last updated by author(s): Apr 10, 2024

Reporting Summary

Nature Portfolio wishes to improve the reproducibility of the work that we publish. This form provides structure for consistency and transparency in reporting. For further information on Nature Portfolio policies, see our Editorial Policies and the Editorial Policy Checklist.

Statistics

For all statistical analyses, confirm that the following items are present in the figure legend, table legend, main text, or Methods section.

n/a	Confirmed	
☐	☒	The exact sample size (n) for each experimental group/condition, given as a discrete number and unit of measurement
☐	☒	A statement on whether measurements were taken from distinct samples or whether the same sample was measured repeatedly
☒	☐	The statistical test(s) used AND whether they are one- or two-sided<br>*Only common tests should be described solely by name; describe more complex techniques in the Methods section.*
☐	☒	A description of all covariates tested
☐	☒	A description of any assumptions or corrections, such as tests of normality and adjustment for multiple comparisons
☐	☒	A full description of the statistical parameters including central tendency (e.g. means) or other basic estimates (e.g. regression coefficient) AND variation (e.g. standard deviation) or associated estimates of uncertainty (e.g. confidence intervals)
☒	☐	For null hypothesis testing, the test statistic (e.g. F, t, r) with confidence intervals, effect sizes, degrees of freedom and P value noted<br>*Give P values as exact values whenever suitable.*
☒	☐	For Bayesian analysis, information on the choice of priors and Markov chain Monte Carlo settings
☒	☐	For hierarchical and complex designs, identification of the appropriate level for tests and full reporting of outcomes
☒	☐	Estimates of effect sizes (e.g. Cohen's d, Pearson's r), indicating how they were calculated

Our web collection on statistics for biologists contains articles on many of the points above.

Software and code

Policy information about availability of computer code

Data collection	Lumerical (2023 v1) used for photonic design, matlab (R2023b) used for data collection and analysis
Data analysis	Lumerical (2023 v1), matlab (R2023b)

For manuscripts utilizing custom algorithms or software that are central to the research but not yet described in published literature, software must be made available to editors and reviewers. We strongly encourage code deposition in a community repository (e.g. GitHub). See the Nature Portfolio guidelines for submitting code & software for further information.

Data

Policy information about availability of data

All manuscripts must include a data availability statement. This statement should provide the following information, where applicable:

- Accession codes, unique identifiers, or web links for publicly available datasets
- A description of any restrictions on data availability
- For clinical datasets or third party data, please ensure that the statement adheres to our policy

The data supporting the findings of this study are available from the article and its Supplementary Information. Due to competing interests with regards to the attempts at the commercialization of this sensor technology by Fingate Technologies Pte. Ltd. and Vulcan Photonics SDN. BHD., the source data is available from the corresponding author upon request.

Research involving human participants, their data, or biological material

Policy information about studies with human participants or human data. See also policy information about sex, gender (identity/presentation), and sexual orientation and race, ethnicity and racism.

Reporting on sex and gender	Not applicable to this study.
Reporting on race, ethnicity, or other socially relevant groupings	Not applicable to this study.
Population characteristics	Not applicable to this study.
Recruitment	Not applicable to this study.
Ethics oversight	Not applicable to this study.

Note that full information on the approval of the study protocol must also be provided in the manuscript.

Field-specific reporting

Please select the one below that is the best fit for your research. If you are not sure, read the appropriate sections before making your selection.

☐ Life sciences ☐ Behavioural & social sciences ☒ Ecological, evolutionary & environmental sciences

For a reference copy of the document with all sections, see nature.com/documents/nr-reporting-summary-flat.pdf

Ecological, evolutionary & environmental sciences study design

All studies must disclose on these points even when the disclosure is negative.

Study description	By leveraging on the integration of crown ethers and silicon photonics, a photonic sensor platform was developed. This photonic sensor technology was tested against various concentrations of Pb2+, across different pH, and field samples.
Research sample	For each concentration of Pb2+, pH, in DI water or field samples, 6 separate and independent sensors (n = 6) was used to analyze the repeatability of the developed sensor technology.
Sampling strategy	The sensor sample size used to measure each concentration of Pb2+, pH, in DI water or field samples were determined via the number of sensors that were fabricated.
Data collection	Specifically, this work shows the development of a photonic sensor platform that facilitates the quantitative detection of Pb2+. First of all, DI water was added into the photonic sensor assembly where the reference resonant wavelength of the sensor was determined. Following, DI water was removed, and the analyte containing Pb2+ ions was added. The photonic sensor is exposed to the analyte-of-interest for 120 s, where it will be flushed out after. Subsequently, DI water was added again into the photonic sensor assembly, where the photonic sensor resonant wavelength is measured again. The extent of wavelength shift between the two resonant wavelengths indicates the concentration of Pb2+ within the analyte, where the concentration can be inferred via the photonic sensor calibration curve. For material characterization, X-ray Photoelectron Spectroscopy (XPS), and Energy Dispersive X-ray (EDX) analysis was used. For the determination of reference Pb2+ concentration in the analyte, Inductively Coupled Plasma Mass Spectrometry (ICP-MS) was used.
Timing and spatial scale	Specifically, this work shows the development of a sensor platform that facilitates the quantitative detection of Pb2+. First of all, DI water was added into the sensor assembly where the reference resonant wavelength of the sensor was determined. Following, DI water was removed, and the analyte containing Pb2+ ions was added. The sensor is exposed to the analyte-of-interest for 120 s, where it will be flushed out after. Subsequently, DI water was added again into the sensor assembly, where the sensor resonant wavelength is measured again. The extent of wavelength shift between the two resonant wavelengths indicates the concentration of Pb2+ within the analyte, where the concentration can be inferred via the sensor calibration curve. For material characterization, X-ray Photoelectron Spectroscopy (XPS), and Energy Dispersive X-ray (EDX) analysis was used. For the determination of reference Pb2+ concentration in the analyte, Inductively Coupled Plasma Mass Spectrometry (ICP-MS) was used.
Data exclusions	No data was excluded.
Reproducibility	An identical photonic sensor testing protocol was utilized across each concentration of Pb2+, pH, in DI water or field samples. For each of the analyte conditions as mentioned above, 6 separate and independent photonic sensors (n = 6) was used to analyze the repeatability of the developed photonic sensor technology. The photonic sensor testing protocol is elucidated as following. DI water was first added into the photonic sensor assembly where the reference resonant wavelength of the photonic sensor was determined. Following, the DI water was removed, and analyte containing Pb2+ ions was added. Thephotonic sensor is exposed to the analyte-of-interest for 120 s, where it will be flushed out after. Subsequently, DI water was added again into the photonic sensor assembly, where the photonic sensor resonant wavelength is measured again. The extent of wavelength shift between the two resonant wavelengths indicates the concentration of Pb2+ within the analyte, where the concentration of Pb2+ can be inferred from the photonic sensor calibration curve.

Randomization	Identical copies of the photonic sensors were fabricated and used for the detection of various concentration of Pb2+, across different values of pH, in DI water or field samples. During the measurement of each analyte condition, there was no specific selection of the photonic sensors.
Blinding	The concentration of Pb2+ within the analyte was determined via ICP-MS first. This enables us to know the reference Pb2+ concentrations. Subsequently, the photonic sensor technology was subjected to analyte with various Pb2+ concentrations, where the accuracy and repeatability in the photonic sensor-inferred concentrations to the reference was analyzed.

Did the study involve field work? ☒ Yes ☐ No

Field work, collection and transport

Field conditions	For the collection of field samples (tap, lake and, water samples) in samples to indicate the relevance of the photonic technology is real samples. The developed sensor technology was subjected to these field samples to detection the concentration of Pb2+. During samples collection, the environmental temperature is about 30 degree Celsius, with humidity around 70 %.
Location	The tap water is collected from the washroom in Nanyang Technology Univeristy, School of Electrical and Electronic Engineering, 50 Nanyang Avenue, 639798, Singapore. The lake and sea water sources are Jurong Lake Garden, 104 Yuan Ching road, 618665, Singapore and West Coast Park Beach, West Coast Ferry Road, 126978, Singapore respectively.
Access & import/export	The abovementioned field samples are collected in compliance with local, national, and international laws.
Disturbance	The collection of the field samples in the described locations do not contribute to disturbance in the environments.

Reporting for specific materials, systems and methods

We require information from authors about some types of materials, experimental systems and methods used in many studies. Here, indicate whether each material, system or method listed is relevant to your study. If you are not sure if a list item applies to your research, read the appropriate section before selecting a response.

Materials & experimental systems

n/a	Involved in the study	
☒	☐	Antibodies
☒	☐	Eukaryotic cell lines
☒	☐	Palaeontology and archaeology
☒	☐	Animals and other organisms
☒	☐	Clinical data
☒	☐	Dual use research of concern
☒	☐	Plants

Methods

n/a	Involved in the study	
☒	☐	ChIP-seq
☒	☐	Flow cytometry
☒	☐	MRI-based neuroimaging

Plants

Seed stocks	Not applicable to this study.
Novel plant genotypes	Not applicable to this study.
Authentication	Not applicable to this study.
